# Intervertebral disc degeneration in mice with type II diabetes induced by leptin receptor deficiency

**DOI:** 10.1186/s12891-020-3091-1

**Published:** 2020-02-05

**Authors:** Xinhua Li, Xiaoming Liu, Yiru Wang, Fuming Cao, Zhaoxiong Chen, Zhouyang Hu, Bin Yu, Hang Feng, Zhaoyu Ba, Tao Liu, Haoxi Li, Bei Jiang, Yufeng Huang, Lijun Li, Desheng Wu

**Affiliations:** 1 Department of Spinal Surgery, Shanghai East Hospital, Tongji University School of Medicine, 150 Jimo Road, Shanghai, 200120 China; 2 Department of Orthopedics, Tongren Hospital, Shanghai Jiao Tong University School of Medicine, 1111 XianXia Road, Shanghai, 200336 China; 3 Department of endocrinology, Shanghai East Hospital, Tongji University School of Medicine, Shanghai, 200120 China

**Keywords:** Diabetes, Leptin receptor, Intervertebral disc degeneration

## Abstract

**Background:**

The leptin receptor-deficient knockout (db/db) mouse is a well-established model for studying type II diabetes mellitus (T2DM). T2DM is an important risk factor of intervertebral disc degeneration (IVDD). Although the relationship between type I diabetes and IVDD has been reported by many studies, few studies have reported the effects of T2DM on IVDD in db/db mice model.

**Methods:**

Mice were separated into 3 groups: wild-type (WT), db/db, and IGF-1 groups (leptin receptor-deficient mice were treated with insulin-like growth factor-1 (IGF-1). To observe the effects of T2DM and glucose-lowering treatment on IVDD, IGF-1 injection was used. The IVD phenotype was detected by H&E and safranin O fast green staining among db/db, WT and IGF-1 mice. The levels of blood glucose and weight in mice were also recorded. The changes in the mass of the trabecular bone in the fifth lumbar vertebra were documented by micro-computed tomography (micro-CT). Tunnel assays were used to detect cell apoptosis in each group.

**Results:**

The weight of the mice were 27.68 ± 1.6 g in WT group, which was less than 57.56 ± 4.8 g in db/db group, and 52.17 ± 3.7 g in IGF-1 injected group (*P* < 0.05). The blood glucose levels were also significantly higher in the db/db mice group. T2DM caused by leptin receptor knockout showed an association with significantly decreased vertebral bone mass and increased IVDD when compared to WT mice. The db/db mice induced by leptin deletion showed a higher percentage of MMP3 expression as well as cell apoptosis in IVDD mice than WT mice (*P* < 0.05), while IGF-1 treatment reversed this situation (*P* < 0.05).

**Conclusions:**

T2DM induced by leptin receptor knockout led to IVDD by increasing the levels of MMP3 and promoting cell apoptosis. IGF-1 treatment partially rescue the phenotype of IVDD induced by leptin receptor knockout.

## Background

Chronic low back pain (LBP) is one of the leading causes of disability in the world, and about 80% of the population experiences chronic LBP at some point of time in their lives [1, 2]. Although the precise causes for chronic LBP are not yet to be determined, intervertebral disc degeneration (IVDD) has been regarded as a major factor for the contribution of LBP [3]. The development of IVDD is a physiologically complex and poorly understood process that involves both environmental as well as genetic factors [4, 5].

According to the previous studies, obesity and type 2 diabetes mellitus (T2DM) have been recognized as two important risk factors for IVDD [6, 7], but their direct association with the condition has never been identified in vivo. Leptin is a 16-kd polypeptide hormone that is encoded by the obese (ob) gene [8, 9]. It is primarily secreted by adipocytes, and functions as an afferent signal in a negative feedback loop through central hypothalamus for regulating adipose tissue mass, energy homeostasis, neuroendocrine function, and metabolism [10]. Mice with mutations in either the leptin gene or in the gene encoding the leptin receptor might develop severe obesity and high glucose levels. The mice with leptin receptor gene knockout (db/db mice) has been extensively used for studying the pathogenesis of T2DM, obesity, leptin signaling, and the interactions among the three [11]. This model was first described in 1965 by Hummel et al. [12], who identified random mutations in mice associated with obesity and excessive hunger. These db/db mice are obese and insulin resistant, with dyslipidemia, and virtually have all mechanistic aspects of T2DM [13] such as neurodegeneration [14], psychiatric disorders [15], osteoarthritis and diabetic-related osteoprosis [16, 17]. Insulin-like growth factor-1 (IGF-1), also called somatomedin C, is a 70-amino acid polypeptide hormone and it plays an important role in normal bone and tissue growth and development [18–20]. IGF-1 administration has been shown to reduce the serum glucose levels not only in healthy individuals, but also in those with insulin resistance, type I diabetes, and T2DM [18], and it is regarded as a promising drug for the treatment of diabetes [18].

Although the db/db mouse model has widely been used to study T2DM, there were few studies that reported the effects of T2MD on intervertebral disc (IVD) in leptin receptor gene knockout mice model and whether it can be rescued by treatment with IGF-1 still unknown. To address these questions, we characterized degenerative changes in the mice IVD, and quantified vertebral bone structure in db/db mice. To observe the effects of T2DM and glucose-lowering treatment on IVDD, IGF-1 injection was used as a glucose-lowering treatment in our study.

## Materials and methods

### Animals

Wild-type male C57BL/6 J mice, and leptin receptor gene knockout, db/db mice were purchased from Shanghai SLAC Laboratory Animal Company Limited, and were housed in the animal facility of Tongji University. Only male mice were included in this study as the female sex steroids protect against the development of diabetes in mice [21–23]. All mice were housed in groups of 2–4 per cage, maintained on a 12-h light/12-h dark cycle, and has unlimited access to food and water for the total study duration. At 5 months of age, db/db mice received intraperitoneal injection of rhIGF-1 0.5 mg/kg every day [24] for 2 months, followed by euthanasia at the age of 7 months.

The blood was collected from the mice for fasting blood glucose analysis before sacrifice. For fasting blood glucose measurement, all the mice are fasted overnight (14 h) and one drop of tail blood was collected for analysis by using a standard glucometer in the morning.

The spines of the sacrificed mice were dissected and immediately frozen in 4% phosphate buffered saline. The adipose tissue was identified and removed by experienced practitioners. All procedures were performed in accordance with the protocol approved by the Institutional Animal Care and Use Committee at Shanghai east hospital.

### Micro-CT skeletal analysis

To quantify the effects of impaired leptin signaling in the spine, the fifth lumbar vertebra from each mouse was scanned using a micro-CT system (MicroCT 40 and vivaCT; ScancoMedical, Basserdorf, Switzerland). A global thresholding procedure was used to separate the calcified tissues from soft tissues. The fifth lumbar vertebrae were then scanned together at 10 μm resolution. The trabecular volumes of interest were outlined by interpolating the operator-drawn regions that exclusively represents the trabecular bone. A 200-slice-thick volume of interest (VOI) was identified at the center of the vertebral body, located at the midpoint between the two endplates. The following parameters were determined for the trabecular bone of the fifth lumbar vertebra: bone mineral density (BMD), relative trabecular bone volume (BV/TV), trabecular thickness (Tb.Th), trabecular spacing (Tb.Sp), and trabecular number (Tb.N). Cortical bone thickness was determined on the ventral cortical wall using the contours of mid-logtitudinal sectional images. Three people performed the radiographic analysis, and all three were blinded to the treatment of animals.

### Histology and immunohistochemistry

Coccygeal (CC) spines were harvested from the mice following euthanasia by using an overdose of pentobarbital (100 mg/kg, IP injection). The coccygeal discs were focused on as they are larger and more readily accessible than lumbar discs. The tails of CC5–6 were then fixed in 4% paraformaldehyde followed by 0.25 M EDTA for decalcification for 3 weeks. The samples were embedded in paraffin, 6 μm sections were prepared by using a standard microtome (RM2255, Leica), and stained with H&E.

Safranin O/fast green staining was performed to visualize the cartilage and assess the proteoglycan content as previously described [16]. Briefly, the regions of interests were manually defined with Image J in order to include the centrally located areas within the morphologically distinct annual fibrosis, endplate, and growth plate regions. The staining intensity was normalized to the background on the same slide and safranin O quantification was performed by using Matlab software. Slides from db/db and IGF-1 mice were normalized to WT mice and only the sections of the same staining procedure were compared.

The sections were prepared and stained with MMP3 antibodies (1:100, sc-21,732, Santa Cruz Biotechnology, CA) for immunohistochemistry. In general, the sections were deparaffinized, rehydrated and treated with 3% hydrogen peroxide in methanol for 30 min for blocking endogenous peroxidase. Antigen retrieval was performed by boiling the samples in a 10 mM citrate buffer (pH 6.0) for 30 min, followed by incubation with MMP3 polyclonal antibody MMP3 for overnight at 4 °C, washed thrice for 5 min each, and then incubated with secondary anti-rabbit antibody for 1 h at room temperature. Negative controls were performed with normal rabbit IgG antibodies (1:100, I-1000-5, Vector lab, USA) under the same conditions. Images were obtained with a Fluo View confocal microscope and prepared with Photoshop software.

### Western blot

Western blotting was performed to detect leptin receptor expression using the leptin receptor antibody (1:400, SAB2700413, Sigma, USA). The whole IVD tissues were dissected respectively from the lumbar and caudal discs of 7 months old mice. The IVD tissues from the 4 mice were pooled in each group. The IVD tissues from the mice were lysed with NP 40 buffer (1% NP-40, 0.15 M NaCl, 50 mM Tris, pH 8.0) containing a protease inhibitor cocktail (PI78441, Fisher Scientific, USA). The cell lysates were centrifuged at 12,000 g for 10 min at 4 °C, and the supernatants were stored at − 80 °C. Protein concentration was measured using BCA protein assay reagent (23,225, Fisher Scientific, USA). Then equal amounts of protein (approximately 20 μg, 1 μg/μl) were denatured in SDS containing Laemmli buffer and separated in 10% SDS–PAGE gels. The proteins were then transferred onto polyvinylidene difluoride membranes (Millipore, USA) in buffer containing 25 mM Tris, 192 mM glycine and 20% methanol. The membranes were blocked with 5% non-fat milk, incubated with a primary antibody for overnight at 4 °C and then incubated with horseradish peroxidase (HRP)-conjugated goat anti-rabbit IgG antibody (1:10,000, A-11034, Novex) at room temperature for 1 h. Enhanced chemiluminescence was performed with Western Bright ECL HRP (Biorad, USA). β-actin (1:2000, sc-47,778, Santa Cruz, USA) was used as an internal control.

### Real-time RT-PCR analysis

All the IVD tissues from a 7 months old mice were dissected respectively from the lumbar and caudal discs. The tissues were subsequently placed in Trizol (15,596,018, Thermo Fisher Scientific, USA). Total RNA was then extracted according to the manufacturer’s instructions, and cDNA was synthesized from 2 μg of total RNA. QPCR was performed with SYBR Green PCR master Mix (B21202, Bimake, USA). All qPCR reactions were run in triplicate and normalized to the expression of GAPDH. The calculation of the relative expression was performed according to the 2-ddCT method. Each reaction was run in triplicate and independently repeated thrice. The sequences and product lengths for each primer pair were as follows: Sox9 (Forward: 5′-TCCCCGCAACAGATCTCCTA-3′; Reverse: 5′-AGGTGGAGTAGAGCCCTGAG-3′;); Aggrecan (Forward: 5′-CGTTGCAGACCAGGAGCAAT-3′; Reverse: 5′-AGGAGTGACAATGCTGCTCA -3′); MMP3 (Forward: 5′-GGCCTGGAACAGTCTTGGC-3′; Reverse: 5′-TGTCCATCGTTCATCATCGTCA-3′); and GAPDH (Forward: 5′-CACATTGGGGGTAGGAACAC-3′; Reverse: 5′- AACTTTGGCATTGTGGAAGG − 3′).

### Tunnel assay

The visualization of apoptotic cells with terminal deoxynucleotidyltranferase-mediated dUTP-biotin nick end labeling (TUNEL) was performed using the paraffin-embedded sections that are analyzed with in situ Cell Death Detection POD Kit (C1056, Beyotime, Shanghai, China). The disc sections from 5 mice in each group (WT group, db/db group, and IGF-1 group) at 7 months of age were used. The apoptotic cells were then counted under 5 visual fields with fluoview software. The TUNEL staining was performed by three researchers who are blinded to the study.

### Statistical analysis

To determine the differences in body weight, blood glucose, microCT data, histology and PCR analysis among the WT, db/db and IGF-1 groups, a 3-way analysis of variance in repeated measures (ANOVA-RM) was performed. All data are presented as means±SD. Significance was set at *P* < 0.05. Each experiment was performed in triplicate.

## Results

### Body composition of mice in each group

The weights of the mice from the three groups were recorded. The mice in the db/db group had significantly higher weights (57.56 ± 4.8 g) than those of the age-matched IGF-1-treated group (52.17 ± 3.7) and the WT group (27.68 ± 1.59 g), (All *P* < 0.05, Table 1). When sacrificed, the db/db mice also had substantially higher fasting glucose concentrations (30.41 ± 3.01 mmol/L) than the WT group (2.46 ± 0.44 mmol/L), and IGF-1-treated group (22.2 ± 2.71 mmol/L), (^*#^*P* < 0.05). The blood glucose levels of over 13.9 mmol/L are regarded as diabetic, and the results showed that the mice in db/db group and IGF-1-treated group had diabetes. The abdominal white adipose tissues were then weighed. In the db/db mice group, the abdominal white adipose tissue was 2.36 ± 0.19 g, out-weighing the 1.75 ± 0.31 g of the IGF-1-treated group (^*^P < 0.05), and the 0.38 ± 0.11 g of the WT group (^*^P < 0.05).

**Table 1:** The body mass, AWATW, and blood gulcose in differnet groups of mice

	WT	IGF-1	db/db mice
Body weight(g)	27.68 ± 1.59	52.17 ± 3.7*	57.56 ± 4.8*a
AWATW(g)	0.38 ± 0.11	1.75 ± 0.31*	2.36 ± 0.19*a
Blood gulcose (mmol/L)	2.46 ± 0.44	22.2 ± 2.71*	30.41 ± 3.01*

*AWATW* Abdominal white adipose tissue weight *P < 0.05(VS WT); a*P* < 0.05(VS IGF-1)

### The bone mass and cortical thinckness of mice in each group

**Fig. 1:**
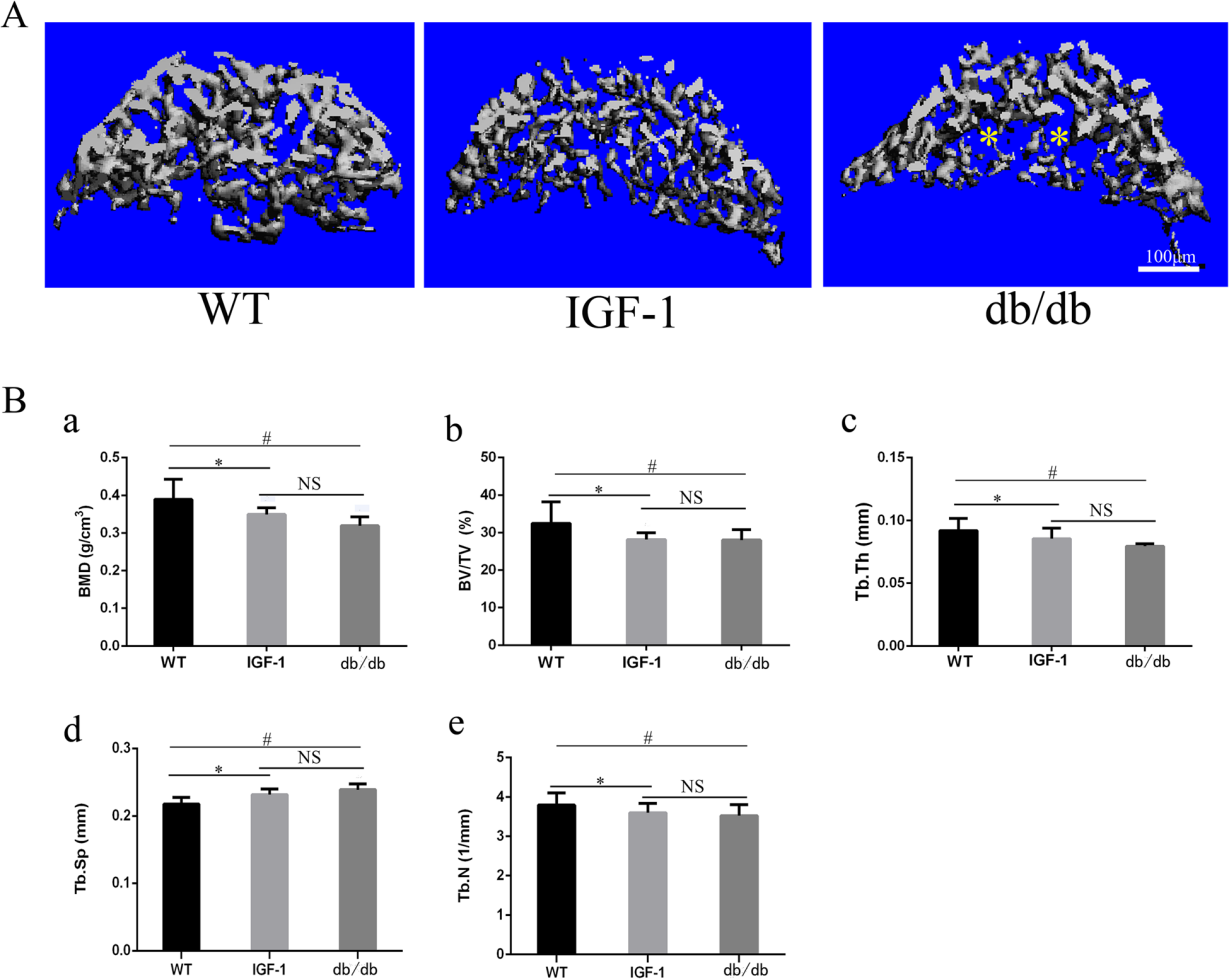
Bone mass decreased in vertebral bone of db/db mice indcued by leptin receptor knock out. (A) Representative μCT scans of the fifth lumbar spine showing the 3D reconstructed trabecular bones. (B) (a) Quantitative analysis of the percentage of bone marrow density (BMD). (b) Quantitative analysis of the percentage of bone volume (BV/TV) (c) Quantitative analysis of trabecular thickness (Tb.Th). (d) Quantitative analysis of trabecular spacing (Tb.Sp). (e) Quantitative analysis of trabecular number (BV/TV). All analyses were performed with 5 mice per group. All data are reported as the mean ± s.d. Statistical significance was determined by three-way ANOVA and Student’s t-test. NS = not significant. ^*^P < 0.05 (IGF-1 group compare to WT). ^#^*P* < 0.05(db/db group compare to WT)

The trabecular bone volume and density in the fifth lumbar vertebra were examined to identify the changes in each group. The BMD, BV/TV, Tb. Sp, Tb.Th and Tb. N were used to evaluate of the bone mass in our study. BMD, BV/TV and Tb. N of the spine showed a significant decrease in db/db and IGF-1-treated mice (^*a^ *P* < 0.05). Tb. Sp and Tb. Th were significantly increased in db/db and IGF-1 mice (^*a^*P* < 0.05). There were no significant differences in terms of BMD, BV/TV, Tb. Sp, Tb.Th and Tb. N in the fifth lumbar vertebra between IGF-1-treated group and db/db mice group (^a^ *P* > 0.05, Fig. 1 and Table 2).

**Table 2:** The lumbar spine trabecular bone volume and density of mice

	WT	IGF-1	DB mice
BMD(g/cm3)	0.39 ± 0.05	0.35 ± 0.02*	0.32 ± 0.02*
BV/TV	32.510 ± 5.769	28.17 ± 1.806*	28.07 ± 2.788*
Tb.Th	0.092 ± 0.009	0.076 ± 0.008*	0.08 ± 0.002*
Tb.sp	0.218 ± 0.009	0.232 ± 0.008*	0.239 ± 0.008*
Tb.N	3.796 ± 0.31	3.404 ± 0.237*	3.428 ± 0.28*

*BMD* Bone mineral density *P < 0.05; a*P* < 0.05(VS IGF-1)

The cortical thickness of the fifth lumbar vertebra were examined to identify changes in the trabecular bone in each group. The cortical thickness of the fifth lumbar vertebra was significantly decreased in db/db and IGF-1-treated mice (^*^P < 0.05). There were no significant differences between the mice of the IGF-1-treated group and db/db mice group (*P* > 0.05), (Additional file 1: Figure S1).

### The results of IVD histological and MMP3 immunohistochemical measurements

**Fig. 2:**
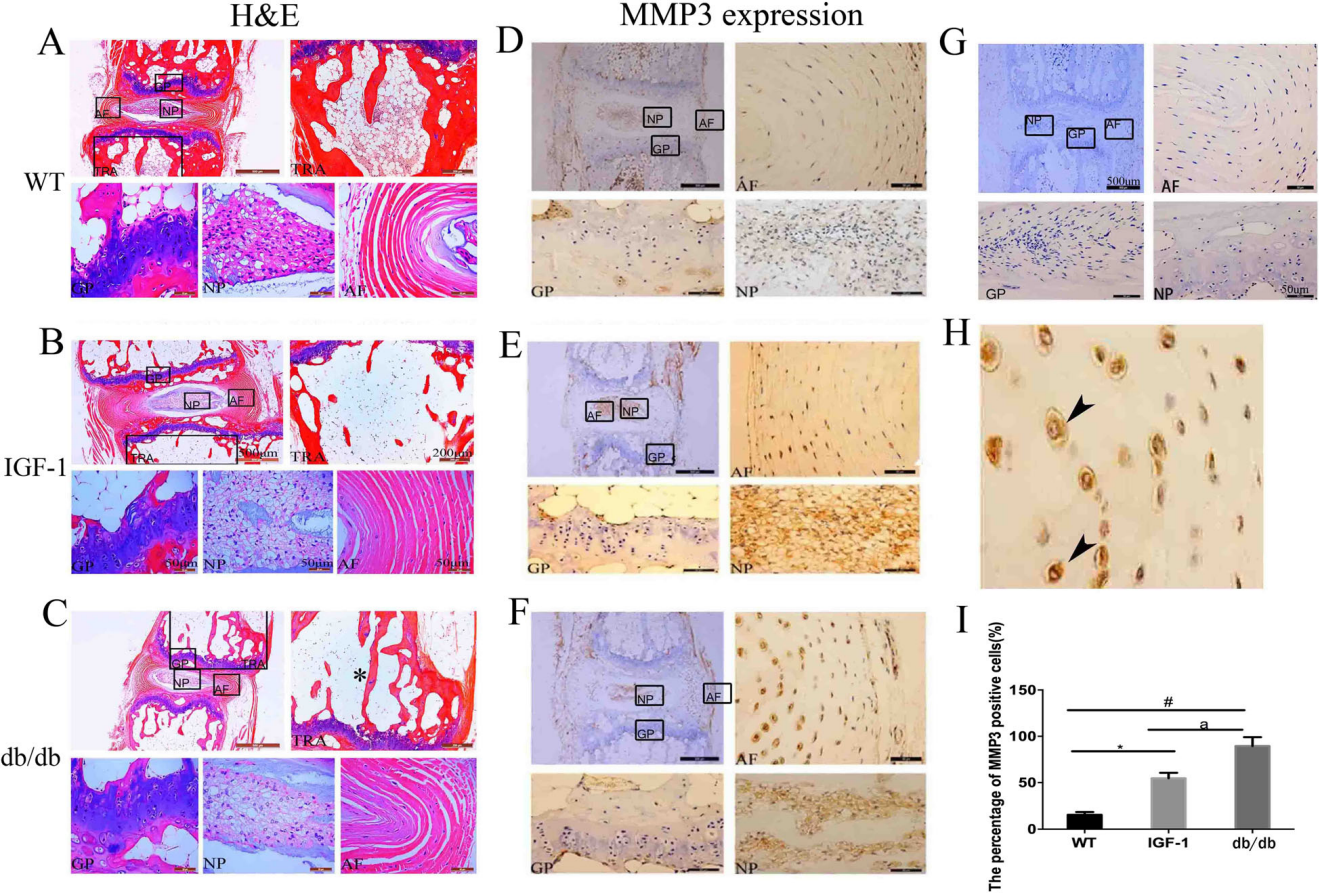
Developed intverterbral disc degenertion phenotype and increased MMP3 expression in intervertebral disc of db/db mice indcued by leptin receptor knock out. **a**, **b**, **c** Representative H&E staining for intervertebral disc in WT, IGF-1 treated and db/db mice; **d**, **e**, **f** Representative picture of MMP3 expression in WT, IGF-1 treated and db/db mice; **g** Representative picture of negative control for MMP3 expression; **h** High mignification picture of MMP3 expression in db/db mice show the MMP3 to be located in extracellular; **i** Quantitative measurements of the percentage of the cells with MMP3 expression to the total cells per view. (*N* = 5, triplicates per group). All data are reported as the mean ± s.d. Statistical significance was determined by three-way ANOVA and Student’s t-test. * *P* < 0.05(IGF-1 treated group compare to WT). #*P* < 0.05(db/db group compare to WT). ^a^*P* < 0.05(IGF-1 treated compare to db/db group)

**Fig. 3:**
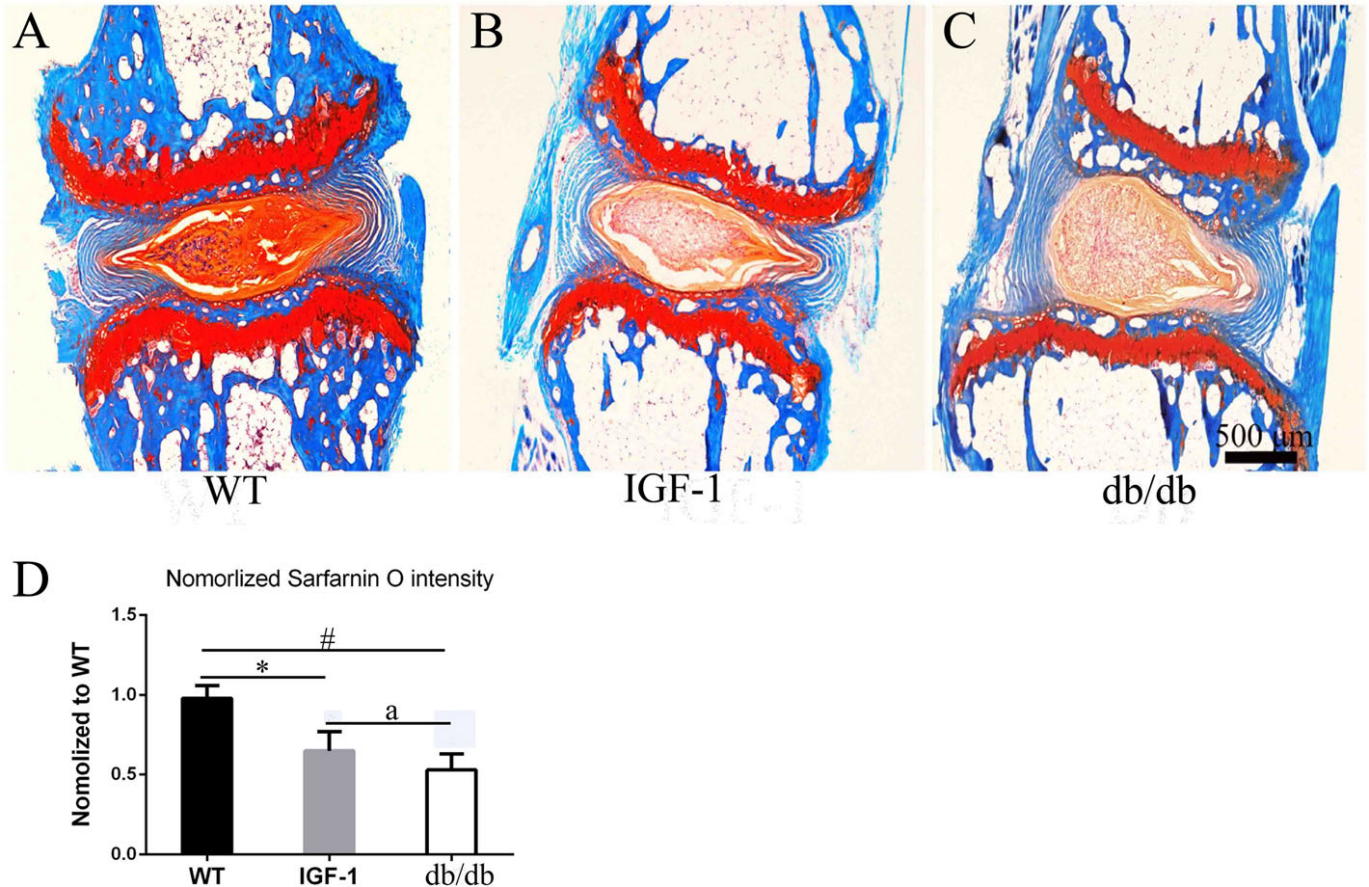
Decreased the extend of Safranin-O staining in intervertebral disc of db/db mice indcued by leptin receptor knock out. **a**, **b**, **c** Representative safranin-O staining of mice intervertebral disc in WT, db/db and IGF-1 treated mice. **d** Quantitative measurements of the extend of Safranin-O staining in intervertebral disc in each group. (N = 5, triplicates per group). All data are reported as the mean ± s.d. Statistical significance was determined by three-way ANOVA and Student’s t-test. ^*^P < 0.05(IGF-1 treated group compare to WT). ^#^*P* < 0.05(db/db group compare to WT). ^a^*P* < 0.05(IGF-1 treated compare to db/db group)

As shown by H&E staining in Fig. 2a, b, c, the db/db mice had significance bone loss in the intervertebral areas of the coccygeal spine. MMP3 expression was shown in extracellular of IVD in all groups mice (Fig. 2d, e , f, g, h. Quantity analysis show that increased MMP3 expression was evident in the IVD of db/db (89%), IGF-1-treated (54%) and WT (15.6%) mice groups. The db/db mice had a much higher MMP3 expression than that of IGF-1-treated mice (^a^*P* < 0.05) (Fig. 2i). Figure 3 shows the representative pictures of safranin-O/fast green staining of mice in each group. The intensity of safranin-O staining (red color) showed a significant decrease in db/db and IGF-1-treated mice when compared to WT mice, while there was no significant difference between db/db and IGF-1-treated mice (Fig. 3d).

### The results of TUNEL staining in each group

**Fig. 4:**
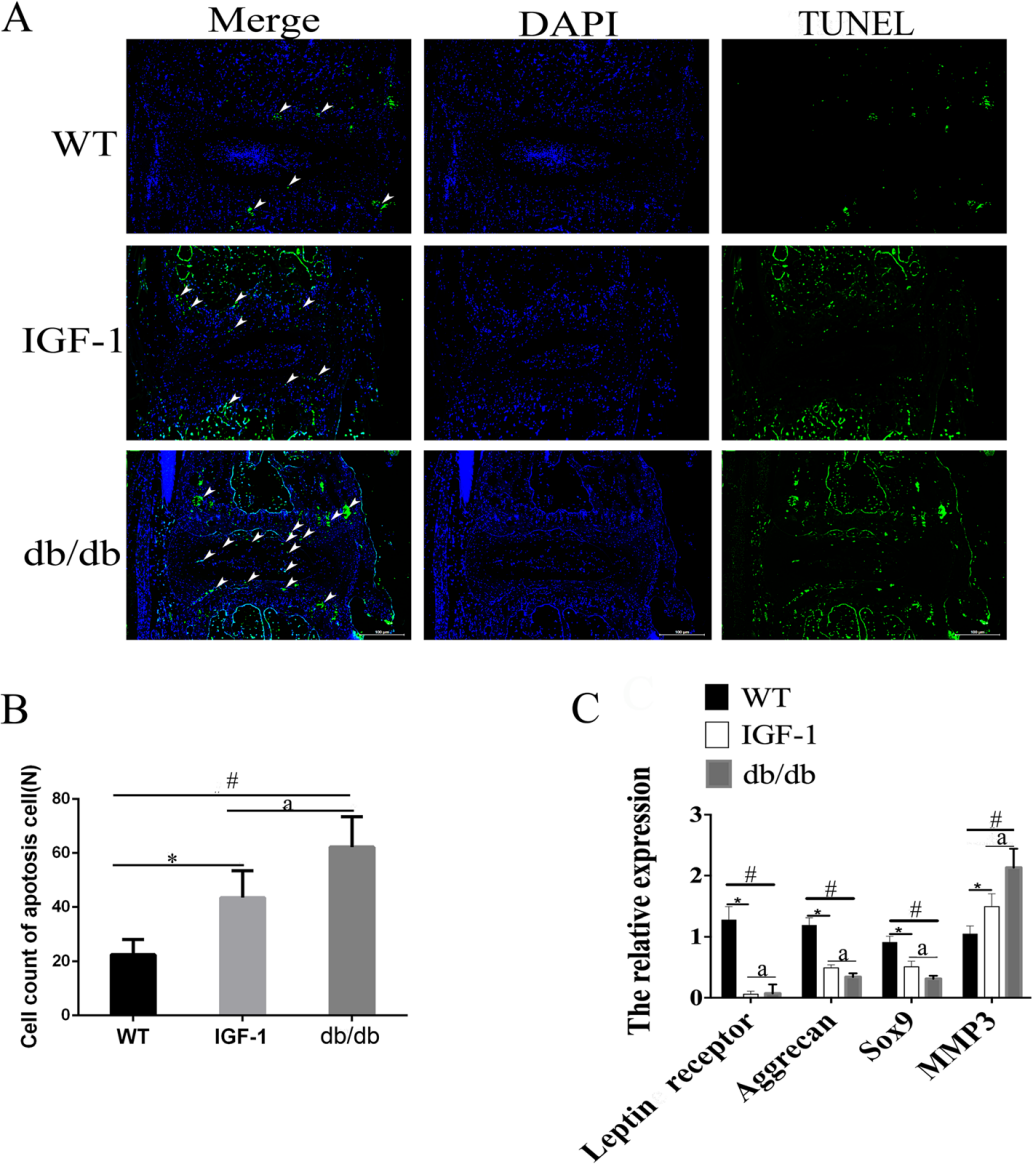
Incresed cell apoptosis in intervertebral disc cells of db/db mice indcued by leptin receptor knock out. **a**, **b**, **c** Representative TUNEL assay shows cell death for the intervertebral disc in WT, IGF treated and db/db group. **d** Quantitative analysis of the percentage of TUNEL-positive cells to the total cells per view in (**a**, **b**, **c**). (N = 5, triplicates per group). All data are reported as the mean ± s.d. Statistical significance was determined by three-way ANOVA and Student’s t-test. ^*^P < 0.05(IGF-1 treated group compare to WT). ^#^*P* < 0.05(db/db group compare to WT). ^a^*P* < 0.05(IGF-1 treated compare to db/db group)

To gain insights into the cellular mechanism of IVD defects caused by leptin receptor deletion, TUNEL staining was performed to detect cell apoptosis. Interestingly, the results revealed a significant increase in the percentage of TUNEL-positive cells in IVDs of db/db and IGF-1-treated mice (Fig. 4a); (^*#^*P* < 0.05). IGF-1 treatment can significantly reduce the percentage of apoptosis in cells (^a^*P* < 0.05).

### The results of gene expression levels in each group

The mouse IVDs from the tail were isolated from WT, IGF-1-treated and db/db mice, and total RNA from these samples was extracted. The gene expression level of leptin was significantly decreased in IGF-1 and db/db groups when compared to those in the controls (^*#^*P* < 0.05), confirming the efficiency of gene deletion (Fig. 4c, Additional file 2: Figure S2). The levels of Sox9, and aggrecan in db/db and IGF-1 group were decreased in IVDs when compared with controls (^a^*P* < 0.05), indicating that leptin receptor is important for maintaining the expression of IVD markers. A significantly increased level of MMP3 in db/db and IGF-1 treated mice indicated the existence of IVDD. When compared to IGF-1-treated mice with db/db mice, significantly increased levels of IVD markers and decreased levels of MMP3 were observed, which indicated that IGF-1 treatment partially rescues the condition of IVDD in db/db mice (^a^*P* < 0.05).

## Discussion

In our study, leptin receptor knockout mice was used as T2DM model to investigate the effects of T2DM on IVDD. The established model allowed us to determine the impact of missing leptin receptors on IVDD and their management, as well as those of T2DM and the glucose-lowering drug IGF-1.

The db/db mice developed a noticeable phenotype as described previously [25]. The weight of the db/db mice reached to 57.56 ± 4.8 g. Both weight as well as size of the mice exceeded those of the age-matched IGF-1-treated mice and WT mice. Our study showed that IGF-1 reduced the abdominal white adipose tissue and weight when compared to those mice in the db/db group. Previous studies showed that the blood glucose levels were also decreased after treatment with IGF-1 [19]. Similar changes in the blood glucose levels showed efficiency of IGF-1-treated group in this study. To determine how impaired leptin receptors and IGF-1 treatment affects lumbar spine, the trabecular bone volume and density were examined in the fifth lumbar vertebra. Both BMD and BV/TV in the spine showed a decrease in the mice of db/db and IGF-1-treated groups, but no significant difference could be detected. The changes in the trabecular bone in the lumbar spines of db/db mice varied in different studies. According to Ducy [10], increased trabecular bone volumes in the vertebrae of db/db mice were confirmed by histology. Several scholars have described increased vertebral length and decreased width in db/db mice [26], but without significant difference in terms of BMD [27] between db/db and WT mice in vertebral. As reviewed by Hamrick [28], increased bone mass in the vertebra bone of db/db mice may reflect different effects of leptin on the axial and peripheral skeleton. However, recent studies have revealed a decrease in bone mass reduction in both peripheral and axial skeletons of db/db mice [25, 29]. In our study, decreased BMD, BV/TV, and Tb. Th were also found in the lumbar spines of db/db mice. Previous studies showed that leptin deficiency has a net negative influence on bone, which is consistent with the direct anabolic effects of leptin. The osteoblast and chondrocyte proliferation and differentiation were directly increased by leptin both in in vitro and in vivo [30–32], while osteoclast formation was inhibited [31–33]. These effects are shown via leptin receptor, in which many groups showed expression of both osteoblasts and chondrocytes [31, 34].

Histology analysis using HE staining, safranin-O/fast green, IHC and TUNEL assay was performed to detect changes in the IVD among the 3 groups. Significance loss of bone mass in the vertebra of coccygeal spine was also found, which was confirmed by micro-CT and H&E staining. These changes may attribute to the extreme fat and high gulcose caused by leptin receptor knockout.

One major change observed in safranin-O/fast green staining is that the extent of sarfarnin O staining decreased in db/db and IGF-1-treated mice in IVD. As known, the area can be stained with red color for safranin-O/fast green staining in IVD due to extensive aggrecan. The loss of red color in NP and GP of IVD indicated that the IVD has much less aggrecan after leptin receptor gene knockout, which confirmded by our PCR detection. The MMP3 was considered as a very important index to evaluate the extent of degeneration of IVD. In our study, increased MMP3 expression was evident in the IVD of db/db and IGF-1-treated groups when compared to WT mice. Db/db mice had a higher percentage of MMP3 than IGF-1-treated mice. TUNEL assay also showed that apoptotic cells were increased in the IVD of db/db and IG-1-treated mice comparing with WT mice. However, comparing with the apoptotic cells in db/db groups, the IGF-1-treated could reduce the percentage of apoptosis cells.

How can IVDD happen in db/db mice? Firstly, leptin can promote the development of disc and cartilage, which has been investigated by several numerous studies [13–17]. Lack of leptin receptors can affect the development of cells in IVD [18]. Secondly, increased inflammation in db/db mice is another important factor to be noted [7]. Inflammation has been recognized as a major contributor of IVDD. Thirdly, high glucose levels and advanced glycation end products (AGE) in db/db mice cannot be ignored.

Obesity and diabetes are subjects of increasing interest in the field of IVDD research [6, 7, 35–40]. T2DM is the most commonly used word globally. Leptin deficient knockout mice are recognized as one of the most widely-used models for studying T2DM recently. Although several studies have investigated the relationship between type 1 diabetes and IVDD, there were only few studies that identified how IVD changes in a T2DM mouse model. In our study, the diabetes condition in leptin receptor knockout mice was confirmed by measuring the fasting blood glucose levels. This was followed by identification of decreased bone mass in the vertebral bone in db/db mice, which in turn mimics osteoporsis or bone mass decrease in patients with T2DM. Epidemiological studies showed that T2DM patients are more prone to develop IVDD, but there is no animal study to investigate how IVD changes under T2DM condition. In our study, we identified that the IVD degenerates in T2DM mice model for the first time. With further investigation of molecular pathological morphology, the levels of MMP3 and apoptosis cell were found to increase significantly. IGF-1 can decrease blood glucose levels and improve the condition of insulin resistance, and it is regarded as a promising drug for treating T2DM [18]. Indeed, IGF-1 treatment can decrease blood glucose levels, partially rescue the extension of degenerated IVD. Intake of blood glucose lowering drug might relieve or rescue IVDD in human patients. However, the bone mass in the vertebral bone did not change after IGF-1 treatment. The treatment time point might be too late or too short to cause increase in the bone mass or remodeling could be a reason.

There are two main explanations as to how IGF-1 can slow down the process of IVDD. Firstly, IGF-1 decreases the blood glucose levels and improves the situation of diabetes [18]. Secondly, IGF-1 is a kind of active polypeptide that inhibits the degeneration of cartilage, promotes the proliferation of chondrocytes, and maintains the stability of cartilage collagen in IVDs [41]. Type II collagen and aggrecan are the main product of the extracellular matrix in the disc [42]. It was reported that IGF-1 can not only stimulates chondrocytes to synthesize type II collagen but also increases the activity of glycosaminoglycans polyenzyme to prevent IVDD. IGF-1 was also reported to promote cells to synthesize aggrecan in the nucleus pulposus [43–45]. All the above points indicate that lack of leptin receptor gene causes loss of bone mass in the vertebra and induces early stage of IVDD, while IGF-1 can salvage the situation to some extent.

However, there are several limitations to this study. Firstly, glucose tolerance test was not performed to verify the diabetic status, and inflammation factors and levels of AGEs were not included in our study. Secondly, although leptin receptor knockout is a most widely used model for T2DM research, there are still some differences between leptin receptor knockout and T2DM in humans, especially for IVD. Finally, the effect of leptin gene knockout on IVD was not taken into consideration.

## Conclusion

This study demonstrated that T2DM induced by leptin receptor knockout led to IVDD by increasing the levels of MMP3 and promoting cell apoptosis. IGF-1 treatment partially rescues the phenotype of IVDD induced by leptin receptor knockout.

## Supplementary information

**Additional file 1: Figure S1.** Decreased cortical bone thinckness in vertebral bone of db/db mice indcued by leptin receptor knock out. (A) Representative μCT scans of the fifth lumbar spine showing the 3D reconstructed cortical bone thinckness in vertebral bone. (B) Quantitative analysis of the cortical bone thinckness in vertebral bone in each group. (*N* = 5, triplicates per group). All data are reported as the mean ± s.d. Statistical significance was determined by three-way ANOVA and Student’s t-test. ^*^P < 0.05(IGF-1 treated group compare to WT). ^#^*P* < 0.05(db/db group compare to WT). NS = not significant.

**Additional file 2: Figure S2.** Leptin receptor expression level dramaticly reduced in intervertebral disc identified by wetstern blot in WT and db/db group.

## Data Availability

All data generated or analysed during this study are included in this published article [and its supplementary information files].

## Abbreviations

DB: Diabetes
IGF-1: Insulin-like growth factor 1
IVDD: Intervertebral disc degeneration
LBP: Lower-back pain
MMP3: Matrix metalloproteinase-3
T2MD: Type II diabetes
WT: Wild type
