# Supplementary figures and images for: Intervertebral disc degeneration in mice with type II diabetes induced by leptin receptor deficiency
Source: BMC Musculoskelet Disord. 2020 Feb 5;21:77. doi: 10.1186/s12891-020-3091-1 (PMC7003448; doi:10.1186/s12891-020-3091-1)

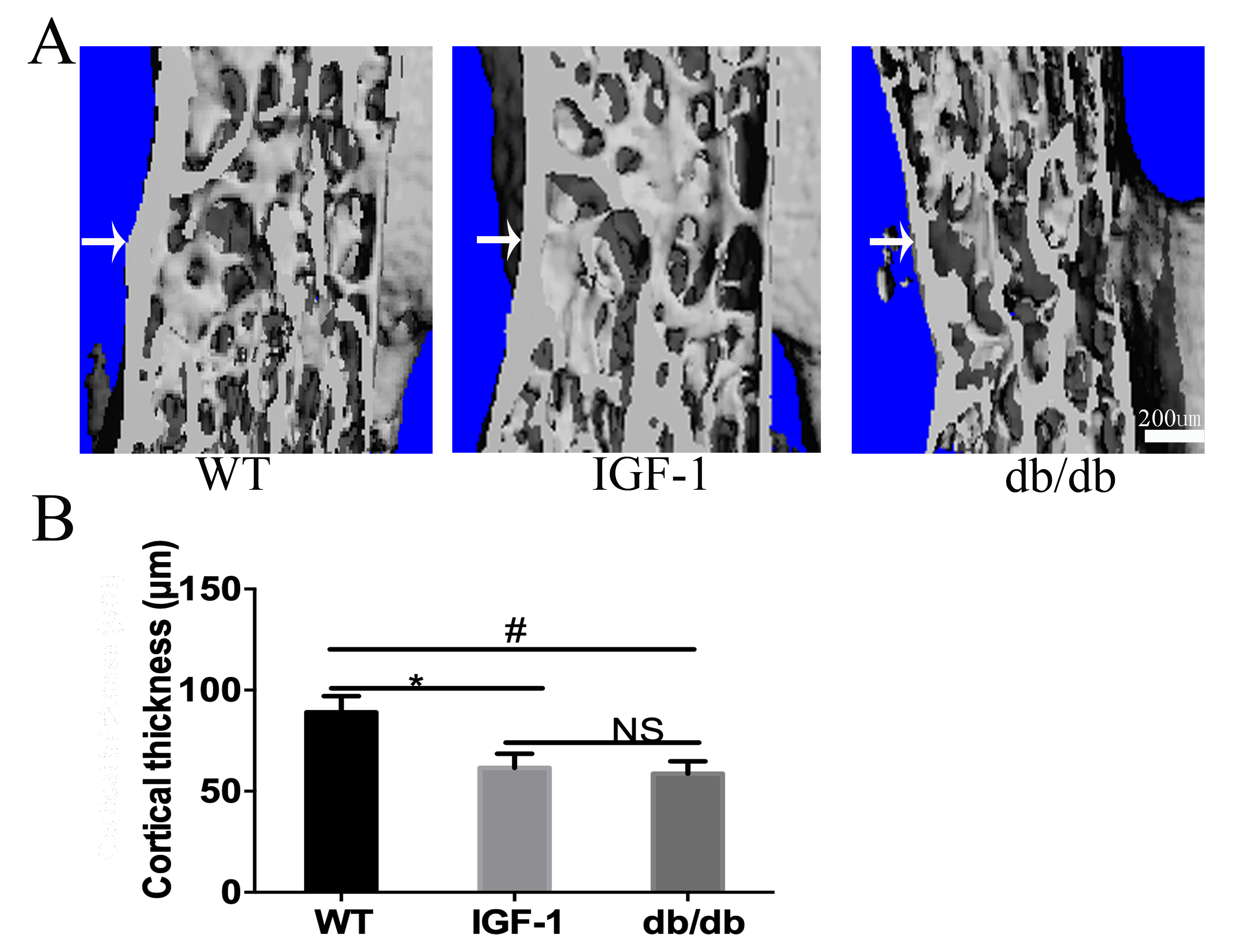

Supplement: Supplementary file 1 — Additional file 1: Figure S1. Decreased cortical bone thinckness in vertebral bone of db/db mice indcued by leptin receptor knock out. (A) Representative μCT scans of the fifth lumbar spine showing the 3D reconstructed cortical bone thinckness in vertebral bone. (B) Quantitative analysis of the cortical bone thinckness in vertebral bone in each group. (N = 5, triplicates per group). All data are reported as the mean ± s.d. Statistical significance was determined by three-way ANOVA and Student’s t-test. *P < 0.05(IGF-1 treated group compare to WT). #P < 0.05(db/db group compare to WT). NS = not significant. [file 12891_2020_3091_MOESM1_ESM.tif]
